# Ion Transport in Electromembrane Systems under the Passage of Direct Current: 1D Modelling Approaches

**DOI:** 10.3390/membranes13040421

**Published:** 2023-04-08

**Authors:** Aminat Uzdenova

**Affiliations:** Department of Computer Science and Computational Mathematics, Umar Aliev Karachai-Cherkess State University, Karachaevsk 369200, Russia; uzd_am@mail.ru

**Keywords:** ion-exchange membrane, electromembrane system, ion transport, galvanodynamic mode, direct current mode, space charge, desalination, mathematical modelling, Nernst–Planck–Poisson equations, displacement current

## Abstract

For a theoretical analysis of mass transfer processes in electromembrane systems, the Nernst–Planck and Poisson equations (NPP) are generally used. In the case of 1D direct-current-mode modelling, a fixed potential (for example, zero) is set on one of the boundaries of the considered region, and on the other—a condition connecting the spatial derivative of the potential and the given current density. Therefore, in the approach based on the system of NPP equations, the accuracy of the solution is significantly affected by the accuracy of calculating the concentration and potential fields at this boundary. This article proposes a new approach to the description of the direct current mode in electromembrane systems, which does not require boundary conditions on the derivative of the potential. The essence of the approach is to replace the Poisson equation in the NPP system with the equation for the displacement current (NPD). Based on the system of NPD equations, the concentration profiles and the electric field were calculated in the depleted diffusion layer near the ion-exchange membrane, as well as in the cross section of the desalination channel under the direct current passage. The NPD system, as well as NPP, allows one to describe the formation of an extended space charge region near the surface of the ion-exchange membrane, which is important for describing overlimiting current modes. Comparison of the direct-current-mode modelling approaches based on NPP and NPD showed that the calculation time is less for the NPP approach, but the calculation accuracy is higher for the NPD approach.

## 1. Introduction

Electromembrane systems are the basis of electrodialysis, nano- and microfluidic devices which are used for water purification, processing of agricultural products (milk, wine, etc.), performing chemical analyses and other types of human activity [1,2,3,4,5,6]. One of the electrical modes in which such systems are operated and investigated is galvanodynamic, that is, the mode where the current density in the system is set. In this case, the current density can be constant (for example, chronopotentiometric studies [7,8,9]), a linear function of time (voltammetry [10]), a periodic function of time (pulsating electric field [11,12], impedance spectroscopy [13]), or another time function.

Mathematical modelling is an important component of membrane systems research, supplementing experimental knowledge. For the mathematical description of the phenomena of mass transfer in electrolyte solutions, several relationships are described in the literature, detailed reviews of which are given in [14,15,16]. The most complete description of transport phenomena in multicomponent electrolyte solutions is based either on the so-called Stefan–Maxwell approach, or on the thermodynamics of irreversible processes, which relates the flows of heat, electricity, momentum, and individual components with the corresponding driving forces in a system of phenomenological equations.

A more simplified approach to describing mass transfer in an electrolyte solution is provided by the Nernst–Planck transfer equation, which takes into account diffusion, migration and convection of ions. The Nernst–Planck equation is derived for dilute solutions and requires only a limited number of parameters, such as diffusion coefficient and ion mobility, which are assumed to be constant. A detailed review of other limitations of the Nernst–Planck equations can be found in [14].

The Nernst–Planck equations are easily combined with other equations describing coupled chemical reactions, concentration polarization and its coupled phenomena [14,15,16,17,18]. The Nernst–Planck equations, together with the Poisson equation for the electric potential, form a system of coupled equations (NPP), which is widely used in studies of electromembrane systems [14,15,16,17,18,19]. This system makes it possible to describe the violation of the electroneutrality of the solution and the formation of a space charge region (SCR) near the membrane surface, due to its permselectivity [19]. The NPP equations, together with the Navier–Stokes equations describing the hydrodynamics of an electrolyte solution near the membrane surface, make it possible to build mathematical models devoted to studying the effect of the SCR and its associated phenomena on the efficiency of mass transfer [20,21].

The electric mode is defined by setting the boundary conditions of the Poisson equation for potential. In the 1D case, to model the potentiodynamic mode at the boundaries of the region under consideration, the values of the electric potential are set, the difference of which is equal to the required voltage. In the galvanodynamic mode, a fixed (for example, zero), potential is set on one of the boundaries, and on the other—a boundary condition on the spatial derivative of the potential. J. Manzanares and co-authors [22] used a 1D system of NPP equations with a boundary condition connecting the derivative of the potential and the given current density to describe the galvanodynamic mode. This boundary condition was obtained by expressing the term of the displacement current from the equation for the total current density. On the basis of this boundary condition, studies were successfully carried out for the galvanodynamic mode of the evolution of the structure of diffusion layers adjacent to the ion-exchange membrane [22], the impedance of the membrane system [23]; the differential capacitance of the electric double layer in the diffusion boundary layer of the ion-exchange membrane was calculated in [24].

In [25], a similar boundary condition was proposed, also obtained from the equation for the total current density, but the spatial derivative of the potential was expressed from the term of the conduction (faradaic) current. Due to the simplicity of implementation of this form of the boundary condition, 2D models were built on it, which made it possible to theoretically study the chronopotentiograms of homogeneous [26,27] and heterogeneous [28] membranes, taking into account the development of electroconvection, as well as to investigate the process of development of the electroconvective flow in the electrodialysis desalination channel under the action of intense direct current [29]. In recent works [12,30], this boundary condition was used to construct a galvanodynamic model of mass transfer in the mode of a pulsating electric field.

When using the boundary condition on the derivative of the potential, the accuracy of the entire calculation is significantly affected by the error in calculating the derivatives of the concentration and potential fields at the boundaries of the area under consideration. The physical essence of the problem of the ion transport in membrane systems is such that the distributions of the concentration of counterions and the potential near the solution/membrane interface are characterized by large gradients (this region is called the quasi-equilibrium part of the EDL), which increase with an increase in the concentration of the electrolyte solution [31]. Therefore, the computational complexity of calculating the NPP equations when such a boundary condition is specified at the solution/membrane interface is much higher than when it is used at the outer edge of the diffusion layer [25].

This article proposes a new approach to modelling ion transport in the electromembrane system in the galvanodynamic mode, taking into account the formation of SCR at intense currents, which does not require setting boundary conditions for the electric potential. The essence of the proposed approach is to replace the Poisson equation in the NPP system with equations for the displacement current (NPD). Mathematical models are constructed and numerical solutions are obtained for the problem of the non-stationary ion transport for the most common variants of the geometry of the system under consideration in the literature: a depleted diffusion boundary layer near the surface of an ion-exchange membrane; a section of the desalination channel from the anion-(AEM) to cation-exchange membrane (CEM). The ion transport models under the direct current based on the NPP and NPD equations are compared in terms of the accuracy of the solution and the calculation time.

## 2. Ion Transport in the Depleted Diffusion Layer near the Surface of the Ion-Exchange Membrane

### 2.1. Mathematical Model

Suppose that in the electrodialysis desalination channel formed between the AEM and CEM there is a laminar flow of a diluted binary electrolyte. Since the main subject of this study is the ways of description of mass transfer, taking into account the formation of an SCR that develops in the electrolyte layer near the membrane surface under the action of an overlimiting direct current, we will consider a depleted diffusion layer formed near the surface of a CEM. The ion-selective properties of the membrane to which the diffusion layer adjoins will be imitated by the boundary conditions.

Assume that the channel is rather short, so that the thickness of the diffusion layer is small compared to the intermembrane distance, and is approximately constant in the tangential direction [14]. Then the mass transfer process can be considered in the direction normal to the membrane surface, without considering the convective transfer (since the flow is laminar). Density, temperature, and the dielectric constant of the solution are considered to be constants; chemical reactions are not taken into account.

**Figure 1:**
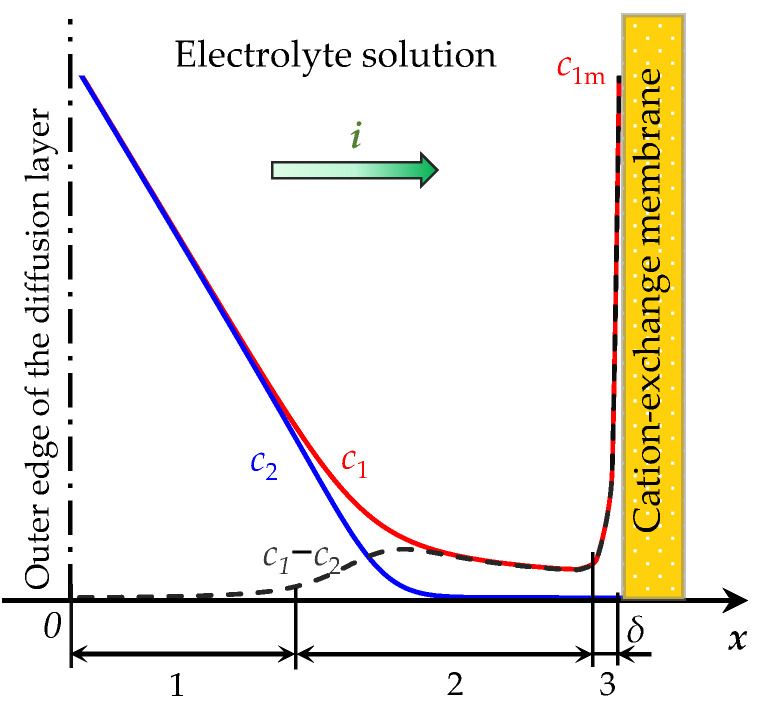
Scheme of the concentration profiles of cations, *c*_1_, anions, *c*_2_, and their difference, *c*_1_ − *c*_2_, in the depleted diffusion layer near the surface of the cation-exchange membrane (CEM). A current of density *i* passes through the system. The regions of the diffusion layer are indicated by numbers: the electrically neutral region (1), the extended region (2) and the quasi-equilibrium region of the space charge (3). *c*_1m_ is the cation concentration at the solution/CEM boundary.

Let *x* be the coordinate normal to the membrane surface, varying from 0 (solution volume, that is, the outer edge of the diffusion layer) to δ (solution/CEM interface), Figure 1. The parameter determining the electric field mode is the current density *i* (*t*).

The mathematical description of the non-stationary ion transport in the dilute electrolyte solution includes the Nernst–Planck equations, Equation (1), the material balance equations, Equation (2), and the Poisson equation for the electric potential, Equation (3), (NPP) [17]. This system of equations for the binary electrolyte in the diffusion layer near the surface of the ion-exchange membrane is written as:

(1) $$j_{n}^{}(x,t)=-\frac{F}{RT}z_{n}D_{n}^{}c_{n}^{}(x,t)\frac{\partial \varphi (x,t)}{\partial x}-D_{n}^{}\frac{\partial c_{n}^{}(x,t)}{\partial x},\;n=1,\;2$$

(2) $$\frac{\partial c_{n}^{}(x,t)}{\partial t}=-\frac{\partial j_{n}^{}(x,t)}{\partial x},\;n=1,\;2$$

(3) $$\varepsilon_{0}\varepsilon_{r}\frac{\partial^{2}\varphi (x,t)}{\partial x^{2}}=-F(z_{1}c_{1}^{}(x,t)+z_{2}c_{2}^{}(x,t))$$

where $j_{n},\;c_{n},\;D_{n},\;z_{n}$ are the flux, molar concentration, diffusion coefficient and charge number of the *n*-th ion, respectively; φ is the electric potential; *ε*_0_ is the electrical constant; *ε_r_* is the relative permittivity of the electrolyte solution (assumed to be constant); *F* is the Faraday constant; *R* is the gas constant; *T* is the absolute temperature. In the NPP system, Equations (1)–(3), the quantities $j_{1},\;j_{2},\;c_{1},\;c_{2},\;\varphi$ are unknown functions of the spatial coordinate *x* and time *t.*

The total current density is described by Equation (4) [32,33]:

(4) $$i_{tot}(t)=i_{F}(x,t)+i_{c}(x,t)$$

where $i_{F}(x,t)=F(z_{1}j_{1}^{}(x,t)+z_{2}j_{2}^{}(x,t))$ is the density of the faradaic current (or conduction current), and $i_{c}(x,t)=-\varepsilon_{0}\varepsilon_{r}\frac{\partial^{2}\varphi (x,t)}{\partial x\partial t}$ is the density of the charging current (or displacement current) associated with the formation and change in the space charge.

Differentiation of the Poisson equation, Equation (3), by time and substitution of the material balance equations, Equation (2), gives the following relation $\frac{\partial i_{F}(x,t)}{\partial x}+\frac{\partial i_{c}(x,t)}{\partial x}=0$. Thus, in the 1D case, the total current density, $i_{tot}(t)$, does not depend on the spatial coordinate *x* and is equal to the given value i(t):

(5) $$i_{tot}(t)=i_{F}(x,t)+i_{c}(x,t)=i(t)$$

Therefore, from the equation for the total current density, Equation (4), it is possible to derive the equation for the electric field strength, E(x,t)=−∂φ/∂x, which simulates the electric mode with a given current density i(t):

(6) $$\varepsilon_{0}\varepsilon_{r}\frac{\partial E(x,t)}{\partial t}=i(t)-F(z_{1}j_{1}^{}(x,t)+z_{2}j_{2}^{}(x,t))$$

Substitution of the Nernst–Planck equations, Equation (1), into the material balance equations, Equation (2), and the equation for the displacement current, Equation (6), gives the closed system of equations for the desired ion concentrations, $c_{1}(x,t)$, $c_{2}(x,t)$, and electric field strength, E(x,t):

(7) $$\frac{\partial c_{n}^{}(x,t)}{\partial t}=-\frac{\partial }{\partial x}\left(\frac{F}{RT}z_{n}D_{n}^{}c_{n}^{}(x,t)E(x,t)-D_{n}^{}\frac{\partial c_{n}^{}(x,t)}{\partial x}\right),\;n=1,\;2$$

(8) $$\begin{array}{l} \varepsilon_{0}\varepsilon_{r}\frac{\partial E(x,t)}{\partial t}=i(t)-\frac{F^{2}}{RT}\left(z_{1}^{2}D_{1}^{}c_{1}^{}(x,t)+z_{2}^{2}D_{2}^{}c_{2}^{}(x,t)\right)E(x,t)+\\ \;\;\;\;\;+F\left(z_{1}D_{1}^{}\frac{\partial c_{1}^{}(x,t)}{\partial x}+z_{2}D_{2}^{}\frac{\partial c_{2}^{}(x,t)}{\partial x}\right) \end{array}$$

Let us abbreviate the system of Equations (7) and (8) as NPD.

The possibility of replacing the Poisson equation with the displacement current equation, which makes it possible to introduce the total current density as one of the independent variables of the problem, was noted by Cohen and Cooley [32]. In [32], the ion transport was calculated based on the Nernst–Planck equations and the displacement current equation, in a completely mechanically permeable membrane, without describing its other physical properties. Brumleve and Buck [33] used this approach to calculate the frequency characteristics of the impedance of a permselective membrane at a constant underlimiting current. In the present work, we use this approach to describe the transport of ions in the depleted diffusion layer near the surface of the ion-exchange membrane.

To solve the NPD system, Equations (7) and (8), boundary conditions are required only for the equations for ion concentrations; the equation for the displacement current density is a spatially distributed ordinary differential equation and requires only initial conditions.

Assume that at the initial time, *t =* 0, no current flows through the system; thus, the electrical neutrality condition is fulfilled at all points of the diffusion layer, and the concentrations of cations and anions are equal to the initial electrolyte concentration, $c_{0}$; the electric field strength is zero:

(9) $$c_{1}\left(x,0\right)=c_{0},\;c_{2}\left(x,0\right)=c_{0},\;E(x,0)=0$$

At the outer boundary of the diffusion layer (*x* = 0), the concentration of both types of ions is fixed and equal to the initial electrolyte concentration, $c_{0}$:

(10) $$c_{1}\left(0,t\right)=c_{0}$$

(11) $$c_{2}\left(0,t\right)=c_{0}$$

According to modern concepts [19,34], when a current flows in the desalination channel, the following ion concentration distribution is formed: the condition of local electrical neutrality is satisfied in the volume of the desalination channel; when approaching the solution/membrane interface, the concentration of counterions passes through a minimum separating the extended SCR and the quasi-equilibrium EDL [19,35] (Figure 1). Within the EDL, the concentration of counterions increases rapidly, and reaches the concentration of fixed ions in the membrane volume. The concentration of co-ions tends exponentially to zero as it approaches the “perfectly selective” membrane. The concentration of counterions at the boundary, *c*_1m_, should be lower than the concentration of fixed ions, but of the same order of magnitude. To find *c*_1m_, several models can be applied, a brief review of which is given in [36].

However, the value of *c*_1m_ does not significantly affect the distribution of concentrations and potential in the extended SCR [35,37]. Therefore, following Rubinshtein and Shtilman [19], we set the concentration of counterions *c*_1m_ under the boundary conditions, and consider it as a parameter. Note that this boundary condition is intensively used in studies of electromembrane systems by Pham et al. [38], Mani et al. [20], Demekhin et al. [39], Shi et al. [40], and others.

In the case of an “ideally selective” membrane, the current is carried exclusively by counterions, and therefore, at the solution/membrane interface, the partial current density of counterions is equal to the total current density, and the normal flux of co-ions is zero. If the permselective properties of the membrane are described using transport numbers of ions, the boundary condition for co-ions is determined based on the equation for the continuity of the ion flux at the solution/membrane interface. Therefore, at the electrolyte solution/membrane interface (*x* = δ), the counterion concentration is set constant, which is $N_{c}$ times greater than the initial electrolyte concentration, $c_{0}$ [19]:

(12) $$c_{1}\left(\delta ,t\right)=N_{c}c_{0}$$

The boundary condition for the concentration of co-ions is formulated using the equation for the continuity of the ion flux at the solution/membrane interface:

(13) $$\left(-D_{2}\frac{\partial c_{2}}{\partial x}-\frac{F}{RT}z_{2}D_{2}c_{2}\frac{\partial \varphi }{\partial x}\right)\left(\delta ,t\right)=\frac{T_{2C}}{Fz_{2}}i(t),$$

where $T_{2C}$ is the effective anion transport number in the CEM. The transport number of an ion in a membrane is defined as the fraction of the conduction current carried by ions of a given species; for example, the transport number of the *n*-th ion in the CEM is equal to $T_{nC}=Fz_{n}j_{n}/i$ [31]. Since the conduction current, $i_{F}$, is realized by ions of both types, the following relations are fulfilled: $T_{1C}+T_{2C}=1$. In ion-exchange membranes placed in dilute electrolyte solutions, the current is carried almost exclusively by counterions, that is, $T_{1C}$ and $T_{2A}$ are close to 1 [41].

Compare the accuracy and required time of calculations based on the NPP and NPD equations. Thus, calculations for the same system parameters will be performed based on two approaches that differ in the way the electric field is described, namely:

(1) The NPD approach, which involves solving the system of the Nernst–Planck and displacement current equations, Equations (7) and (8), with boundary conditions (9)–(13);

(2) The NPP approach, which involves solving the system the Nernst–Planck–Poisson equations, Equations (1)–(3). In this case, conditions for the electric potential are added to the boundary conditions (9)–(13). The system of Equations (1)–(3) includes the potential of the electric field only in the form of spatial derivatives, and therefore only the potential drop, φ(δ,t)−φ(0,t), is significant. For the convenience of calculations, the zero potential is set on the right boundary, x=δ:

(14) φ(δ,t)=0.

The condition, which introduces the density of the flowing current into the problem formulation of the NPP approach [25], is placed on the left boundary, x=0:

(15) $$\frac{\partial \varphi }{\partial x}\left(0,t\right)=-\frac{RT}{F^{2}}\left(\frac{i(t)+Fz_{1}D_{1}\frac{\partial c_{1}(0,t)}{\partial x}+Fz_{2}D_{2}\frac{\partial c_{2}(0,t)}{\partial x}}{z_{1}^{2}D_{1}c_{1}(0,t)+z_{2}^{2}D_{2}c_{2}(0,t)}\right)$$

The initial condition on the potential sets the zero value in the entire diffusion layer:

(16) φ(x,0)=0

### 2.2. System Parameters

The values of the parameters of the membrane system, which are typical for real chronopotentiometric experiments, were chosen, namely: intermembrane distance *H* = 5.67 × 10^−3^ m, channel length *L* = 20 × 10^−3^ m, the temperature *T* = 293 K, concentration of NaCl electrolyte solution *c*_0_ = 10 mol/m^3^, the average velocity of forced flow *V*_0_ = 3.8 × 10^−3^ m/s. These values of the parameters make it possible to estimate the thickness of the diffusion layer using the Leveque formula, δ = (*H*/1.47) (*LD*/(*H*^2^*V*_0_))^1/3^ [42]. For the indicated parameters, thickness of the diffusion layer δ ≈ 0.247 × 10^−3^ m. It is also necessary to determine the diffusion coefficients of cations *D*_1_ = 1.33 × 10^−9^ m^2^/s and *D*_2_ = 2.05 × 10^−9^ m^2^/s; the ion charge numbers *z*_1_ = 1, *z*_2_ = −1. To simplify the numerical solution, the ratio of the counterion concentration at the solution/CEM interface to its value in the solution volume was taken to be *N_c_* = 1. This value is less than in real systems [19]; however, it was shown in [35] that for *N_c_* ≥ 1, the value of *N_c_* does not significantly affect the distribution of concentrations and potential in the extended SCR. Consider the direct current mode with the density *i* = 2*i*_lim_, where *i*_lim_ is the limiting current density determined by Equation (17) [42]:

(17) $$i_{\lim}=\frac{FDc_{0}}{H(T_{1C}-t_{1})}\left[1.47{\left(\frac{H^{2}V_{0}}{LD}\right)}^{1/3}-0.2\right]$$

where $D=D_{1}D_{2}\left(z_{1}-z_{2}\right)/\left(D_{1}z_{1}-D_{2}z_{2}\right)$ is the diffusion coefficient of the electrolyte, $t_{1}$ = 0.395 is the transfer numbers of cation in the solution.

The boundary value problems of the considered mathematical models were solved by the finite element method using the Comsol Multiphysics 6.1 package (www.comsol.com, (accessed on 14 March 2023) COMSOL AB, Stockholm, Sweden).

The computational complexity of the considered problem of describing the ion transport in the diffusion layer near the membrane surface is associated with large gradients of ion concentrations and electric potential in the quasi-equilibrium part of the EDL (Figure 1). The computational complexity increases rapidly as the thickness of this region decreases, which is estimated to be of the order of the Debye length $L_{D}=\sqrt{\varepsilon \varepsilon_{0}RT/\left(2c_{0}F^{2}\right)}$ [31]. The latter occurs with an increase in electrolyte concentration $c_{0}$. Therefore, to perform calculations for concentration values corresponding to the values used in real experiments, a non-uniform computational mesh is construct. Namely, the area under consideration is divided into two parts:

- Region I is the main part of the diffusion layer (with the exception of the thin layer at the solution/membrane interface, it is $100L_{D}$ thick), with a uniform distribution of elements. The main control parameter of the computational mesh in this region is the number of elements (a series of values 1000, 2000, …, 6000 is set).
- Region II is a layer of $100L_{D}$ thickness at the solution/membrane interface, with a linearly decreasing size of computational mesh elements. The main control parameters of the computational mesh in this area are the number of elements (set to 400) and the ratio of the length of the first element to the last (set to 1000).

For the considered parameters, the Debye length is $L_{D}=3.06\times 10^{-9}$ m.

Thus, the calculations based on the NPP and NPD approaches, performed with the same system parameters, solver tolerance settings on the same computational mesh, will be compared.

### 2.3. Results and Discussion

**Figure 2:**
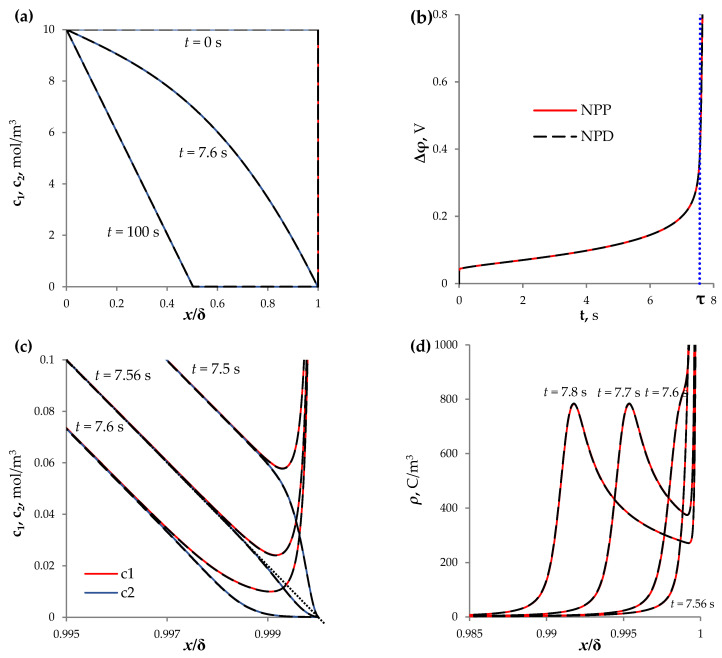

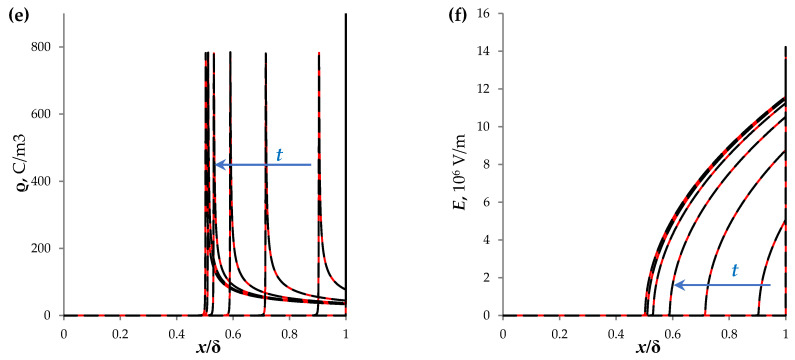
(**a**) Concentration profiles of cations, *c*_1_, and anions, *c*_2_, in the diffusion layer near the CEM surface at *t* = 0, 7.6, 100 s; (**b**) chronopotentiograms; (**c**) distribution of concentrations *c*_1_ and *c*_2_ in the area near the surface of the CEM at *t* = 7.5, 7.56, 7.6 s; (**d**) distribution of space charge density, ρ = *F*(*z*_1_*c*_1_ + *z*_2_*c*_2_), at *t* = 7.56, 7.6, 7.7, 7.8 s; (**e**) distribution of space charge density, ρ, at *t* = 5, 10, …, 30, 100 s; (**f**) distribution of electric field strength, *E*, at *t* = 5, 10, …, 30, 100 s. The results of calculations by NPP (red and blue solid lines) and NPD (dashed black lines) approaches are shown.

Figure 2a shows the concentration profiles at *t* = 0, 7.6, 100 s, calculated based on the NPP and NPD approaches.

At the initial time (*t* = 0 s), the uniform distribution of the concentration of cations and anions (which are equal to $c_{0}$, according to the initial condition (9)) determines the initial resistance of the electrolyte solution. On the chronopotentiogram (ChP, that is, the dependence of the potential drop on time at a constant current density), the initial almost vertical section is observed (Figure 2b, *t* ≈ 0 s).

Over time, the electrodiffusion process reduces the ion concentration near the membrane surface. The formation of concentration gradients in the electrolyte, due to the passage of current through its interface with the ion-selective surface, is called concentration polarization [18]. An increase in concentration polarization reduces the conductivity of the solution, which limits the rate of mass transfer. Therefore, there is a segment of slow growth of the potential drop on the ChP (Figure 2b, 0 < *t* < τ). At *t* = τ = 7.56 s, the tangent to the concentration profiles (in the electrically neutral part) tends to 0 at *x* = δ (Figure 2c).

Furthermore, an extended space charge region (SCR) begins to form at the outer edge of the EDL (Figure 2c,d), and the rapid increase in the potential drop is noted on the ChP (Figure 2b, *t* > τ). The local maximum of the space charge density, $\rho =F(z_{1}c_{1}^{}+z_{2}c_{2}^{})$, is shifted into the volume of the solution (Figure 2d,e). The formation of the extended SCR increases the electric field strength in this region (Figure 2f). Over time, the system passes into the stationary state (with the constant potential drop), in which the local maximum of the space charge is located almost in the middle of the diffusion layer (Figure 2e).

It should be noted that there is good agreement (a difference less than 0.3%) between the time, τ = 7.56 s, and the analytic assessment of the Sand transition time, $\tau_{S}$, determined by Equation (18) [43]:

(18) $$\tau_{S}=\frac{\pi D}{4}{\left(\frac{c_{0}Fz_{1}}{T_{1}-t_{1}}\right)}^{2}\frac{1}{i^{2}}$$

Equation (18) was derived from the theoretical analysis of the infinite diffusion layer, and determines the moment when the electrolyte concentration on the membrane surface reaches 0 [43].

The described features of the mass transfer process in the diffusion layer near the membrane surface are in agreement with the modern ideas about this process that have been developed in [19,22].

In our work [26], a comparison was made between the ChPs obtained experimentally [44] and theoretically, using the NPP model. The experiment [44] was carried out with a laboratory cation-exchange membrane MK-40_MOD_ with an electrically homogeneous surface and 0.02 M (20 mol/m^3^) NaCl solution at *i*/*i*_lim_ = 1.7. Good agreement between the experimental and calculated curves is observed at times *t* < τ_S_. As t approaches τ_S_, the theoretical curve rises steeply, while the experimental curve also rises, but not so steeply; it slows down and forms an inflection point, then flattens out and reaches a steady state. The slowdown of the ChP is usually associated with the development of current-induced convection, which under the experimental conditions is electroconvection [44]. Electroconvection mixes the solution at the surface: it provides additional delivery of a more concentrated electrolyte from the volume of the solution to the surface, and pumps out the depleted solution from the near-surface region [20,21]. Thus, for a more accurate description of ChPs in systems with ion-exchange membranes, the proposed one-dimensional modelling (both NPP and NPD) should be extended at least to a two-dimensional description with the addition of the Navier–Stokes equations, which take into account the effect of electric force on the spatial electric charge in solution.

**Figure 3:**
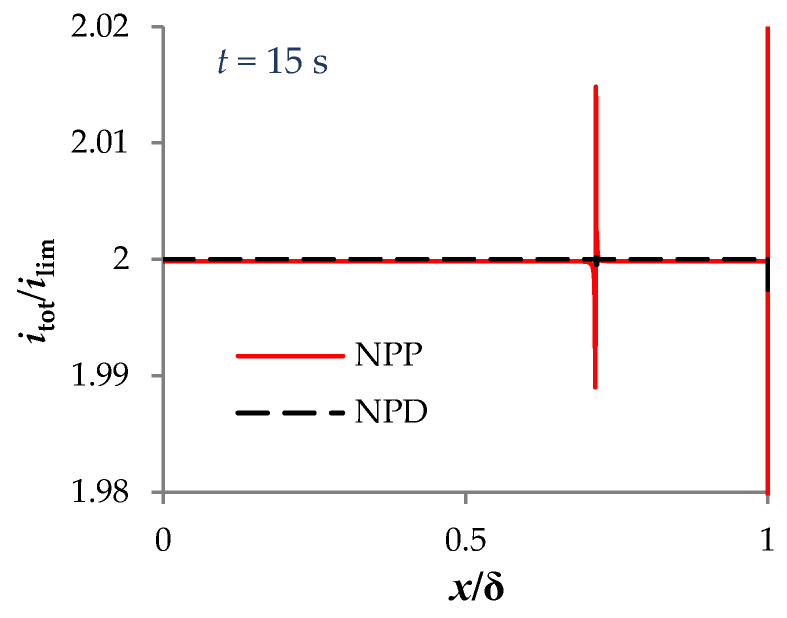
Distribution of the total current density at *t* = 15 s. The results of calculations using NPP (red solid line) and NPD (black dashed line) approaches are shown.

Let us estimate the error in calculating the characteristics of mass transfer in a diffusion layer with the direct current flow based on the NPP and NPD approaches. The calculation error can be estimated from the error in fulfilling Equation (5), since the total current density in the 1D case at each point of the region under consideration must be equal to the given current density. The computational complexity of calculating fields with large gradients leads to the increase in the error of the current density calculation in the quasi-equilibrium part of the EDL and in the vicinity of the local maximum of the extended SCR. For example, Figure 3 shows the distribution of the total current density at *t* = 15 s: significant deviations of the current density from the specified value 2*i*_lim_ are observed at the right boundary, x=δ, and in the region of the local maximum of the extended SCR, x≈0.72 δ. Therefore, the calculation error is determined in the main part of the diffusion layer (Region I):

(19) $$r_{I}(t)=\underset{x\in \left[0,\delta -100L_{D}\right]}{\max}\frac{\left|i_{tot}(x,t)-i\right|}{i}$$

and in the quasi-equilibrium part of the EDL (Region II):

(20) $$r_{II}(t)=\underset{x\in \left(\delta -100L_{D},\delta \right]}{\max}\frac{\left|i_{tot}(x,t)-i\right|}{i}$$

**Figure 4:**
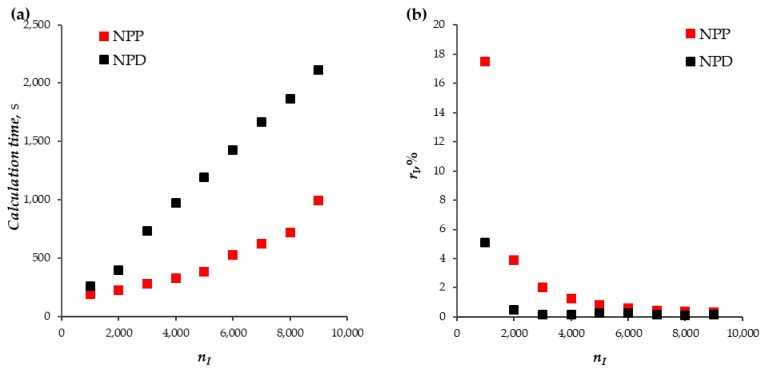

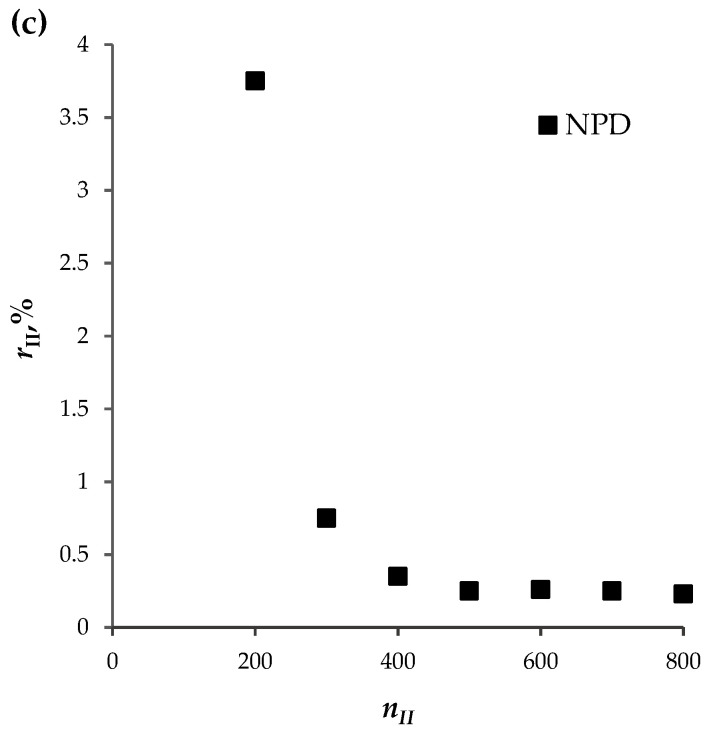
(**a**) Time taken to calculate the model for *t* from 0 to 100 s; (**b**) calculation error in the region I (main part of the diffusion layer), $r_{I}$; (**c**) calculation error in the second region, $r_{II}$. The results of calculations using NPP (red markers) and NPD (black markers) approaches are shown. $n_{I},\;n_{II}$ are the number of elements in regions I and II, respectively.

A series of calculations based on the NPP and NPD approaches has been performed for the following values of the parameter of the computational mesh: the number of elements in the main part of the diffusion layer (Region I) $n_{I}$ = 1000, 2000, …, 9000. Figure 4a,b show the values of the total time spent on the calculation of the model for t from 0 to 100 s and the maximum value of the calculation error in the main part of the diffusion layer in this time interval, $r_{I}=\underset{t\in \left[0,\;100s\right]}{\max}r_{I}(t)$. The time step is automatically selected by the solver, taking into account the set relative-tolerance value. The calculations presented here were performed with the relative-tolerance value equal to $10^{-6}$, which made it possible to achieve an error of less than 1%.

The calculation error $r_{I}$ of the NPP approach is greater than that of the NPD approach for all considered values of the number of mesh elements. Note that for the NPP approach, the condition $r_{I}<1\%$ is satisfied with the number of elements equal to 5000 or more, and for the NPD approach, at 2000 or more (Figure 4b). The calculation time of the NPP approach is on average 2.4 times less than that of the NPD approach (Figure 4a).

It was possible to achieve the calculation error of the current density in the Region II, $r_{II}$, of less than 1% by increasing the number of elements in this region only for the NPD approach (starting from $n_{II}$ = 300 and more), Figure 4c. For the considered concentration of the solution, $c_{0}=10$ mol/m^3^, the order of the calculation error of the current density in the Region II based on the NPP approach was $10^{3}$% (therefore, these data are not shown in Figure 4c); only for small concentrations ($c_{0}\leq 0.01$ mol/m^3^) can an acceptable error be obtained. To overcome this problem of the NPP approach, a numerical–analytical method was proposed in [25], according to which the solution is obtained by a combination of the numerical solution in the electrically neutral region and extended SCR, with the analytical solution in the quasi-equilibrium EDL.

In the NPP approach, the boundary condition (15), which determines the galvanodynamic mode, is set at the outer edge of the diffusion layer (x=0), on which the current density is calculated with high accuracy (Figure 3). Therefore, despite the low accuracy of the current density calculation in the NPP approach at the solution/membrane interface (x=δ), the ChPs calculated based on the NPP and NPD approaches practically coincide (the difference is less than 0.27%). However, for the problem of describing mass transfer in a desalination channel for the galvanodynamic mode, the described problem of the NPP approach limits its direct application to only the range of low electrolyte concentrations [45]. This is due to the fact that, in this case, both boundaries of the region under consideration are solution/membrane boundaries: on one side with the AEM, on the other side with the CEM.

## 3. Ion Transport in the Section of the Desalination Channel

### 3.1. Mathematical Model

The high accuracy of calculation at the solution/membrane interface of the NPD approach makes it possible to solve the problem of describing the ion transport in the desalination channel under the direct current for the values of electrolyte concentrations used in experimental studies. The model for the section of the desalination channel differs from the model for the diffusion layer in the following elements:

- The geometry of the model is the segment with the length equal to the intermembrane distance, *H*;
- Near the solution/AEM boundary (x=0), the $100L_{D}$ -thick region with a linearly increasing size of computational mesh elements (the number of elements is set to 400 and the ratio of the length of the first element to the last one is equal to 1000), is selected;
- The boundary conditions at x=0 are replaced by the conditions:
  (21) $$\left(-D_{1}\frac{\partial c_{1}}{\partial x}-\frac{F}{RT}z_{1}D_{1}c_{1}\frac{\partial \varphi }{\partial x}\right)\left(0,t\right)=\frac{T_{1A}}{Fz_{1}}i(t),$$
  (22) $$c_{2}\left(0,t\right)=N_{a}c_{0}$$
  where $T_{1A}$ is the effective transfer number of cations in the AEM (taken as $T_{1A}$ = 0.972); $N_{a}$ is the ratio of the anion concentration at the solution/AEM interface to its value in the bulk solution (taken as $N_{a}$ = 1).

### 3.2. System Parameters

Calculations for the section of the desalination channel were performed with the system parameters given in Section 2.2.

### 3.3. Results and Discussion

**Figure 5:**
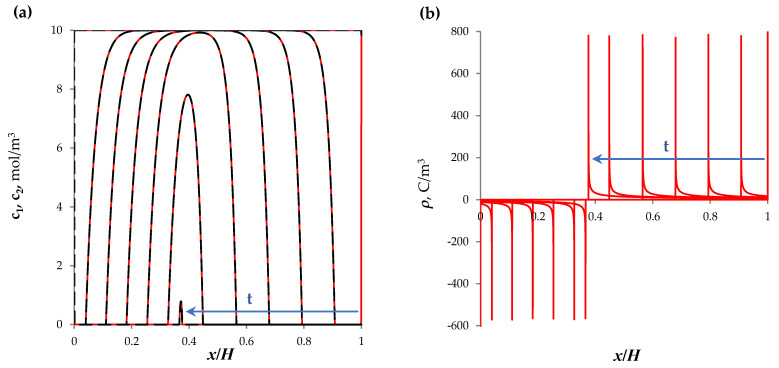
(**a**) Concentration profiles of cations, *c*_1_, (solid lines) and anions, *c*_2_, (dashed lines) in the section of the desalination channel; (**b**) distribution of the space charge density, ρ. The results of calculation using the NPD approach at *t* = 0, 50, …, 250, 268 s are shown.

Figure 5 shows the concentration profiles (Figure 5a) and the charge density distribution (Figure 5b) for the section of the desalination channel with the quiescent electrolyte solution under the action of direct current at *t* = 0.50, …, 250, 268 s. At *t* = 268.15 s, the ion concentration is completely depleted, which coincides with the analytical estimate of this time, $\tau_{b}$, obtained in [45]:

(23) $$\tau_{b}\approx \frac{z_{1}Fc_{0}h}{i(1-T_{1A}-T_{2C})}$$

## 4. Conclusions

New mathematical models of the ion transport in the diluted diffusion layer near the surface of the ion-exchange membrane and in the section of the desalination channel between AEM and CEM under the passage of direct current are constructed, based on the NPD equations. These models make it possible to describe the violation of the electrical neutrality of the solution and the formation of the extended SCR under the action of an overlimiting direct current. The solutions obtained on the basis of NPD are in good agreement with the analytical estimates of the transition time for the diffusion layer and the time of complete desalting of the quiescent electrolyte solution in the desalination channel formed between the AEM and the CEM. 

The comparison of the new approach to modelling the direct current mode based on the NPD equations with the previously described approach based on the NPP equations is made. It is shown that, although the calculation time of the NPD approach is longer than of the NPP, it is characterized by a smaller calculation error.

For a more accurate description of ChPs in systems with ion-exchange membranes, the proposed 1D modelling should be extended to a 2D description with the addition of the Navier–Stokes equations, where the effect of the electric force on the electric charge is taken into account. The development of the proposed approach for multi-species systems, which will be processed in real-world systems with ion-exchange membranes, is also planned in the subsequent work.

## Data Availability

Not applicable.
